# Influence of Laser-Welding on Microstructure and Corrosion Properties of Twinning-Induced Plasticity (TWIP) Steel

**DOI:** 10.3390/ma13194315

**Published:** 2020-09-28

**Authors:** Chengcheng Xu, Youkang Zhang, Wanlei Liu, Ying Jin, Lei Wen, Dongbai Sun

**Affiliations:** 1 National Center for Materials Service Safety, University of Science and Technology Beijing, Beijing 100083, China; hotxcc@126.com (C.X.); Liuwanlei_2020@126.com (W.L.); yjin@ustb.edu.cn (Y.J.); 2 Beijing Aerospace Xinli Technology Co., Ltd., Beijing 100039, China; youkangz@163.com; 3 Innovation Group of Marine Engineering Materials and Corrosion Control, Southern Marine Science and Engineering Guangdong Laboratory, Zhuhai 519080, China; 4 School of Materials, Sun Yat-sen University, Guangzhou 510275, China

**Keywords:** TWIP steel, laser welding, electron back-scattering diffraction EBSD, corrosion property, scanning Kelvin probe (SKP), surface potential

## Abstract

The effect of welding speed on microstructure, mechanical properties, and corrosion properties of laser-assisted welded joints of a twinning-induced plasticity (TWIP) steel was investigated by using X-ray diffraction (XRD), scanning electron microscopy (SEM), electron backscattered diffraction (EBSD) analysis, electrochemical test, and micro-area scanning Kelvin probe test (SKP). The results reveal that the welded joints, with a fully austenitic structure, are obtained by laser welding. In addition, the preferred orientation of grains in fusion zone (FZ) increased with the increase of welding speed. Additionally, the coincidence site lattice (CSL) grain boundaries of FZ decreased with increasing welding speed. However, potentiodynamic polarization and SKP results demonstrated that the welding speed of 1.5 m/min renders superior corrosion resistance. It can also be inferred that the corrosion properties of the welded joints are related to the grain size and frequency of CSL grain boundary in FZ.

## 1. Introduction

The effect of twinning-induced plasticity (TWIP) has been developed to fabricate advanced high-strength steel, where mechanical deformation is introduced to produce twins. TWIP steel renders a high tensile strength of >600 MPa, an extremely high elongation of ~95% and superior toughness. The combination of high strength, high plasticity, and high strain hardening makes TWIP steel an ideal candidate for automobiles [1,2,3,4,5,6].

Laser welding has the characteristics of high energy density, high efficiency, large temperature gradients, high repeatability of the process, and formation of a narrow heat affected zone (HAZ), which can significantly improve welding accuracy and production efficiency in actual processing [7,8,9]. In recent years, the research on laser welding of TWIP steel has mainly focused on the structure and mechanical properties of welded joints under welding process [10,11,12]. The microstructure and mechanical properties, i.e., strain rate [13], strain hardening behavior [14], fatigue fracture [15], temperature and stacking fault energy [16,17], and crystallographic behavior [18], of high-manganese TWIP steel have been widely investigated.

As a steel plate for automobiles, TWIP steel serves in various corrosive environments, such as salt spray and dust in humid environments, antifreeze salt in cold environments, etc. Herein, the corrosion property of TWIP steel is worthy of attention. In addition, the effect of grain structure [19,20], alloying elements [21,22], stress corrosion cracking [22,23], and heat treatment process [24] on corrosion properties of TWIP steels have been studied.

Moreover, as the weakest part of TWIP steel components, the welded joint determines the lifetime of TWIP steel components in harsh working environments [25,26]. Due to the unevenness of the structure, the welded joint is sensitive to corrosion, especially galvanic corrosion [27,28]. However, the corrosion behavior of TWIP steel laser-welded joints has rarely been investigated, as no relevant reference has been found. Therefore, it is very necessary to establish the relationship between the microstructure and the corrosion properties in the micro-area.

The microstructure and corrosion properties for laser-welded TWIP steel, with different welding speeds, have been studied in detail to explore the influence of laser welding speed on corrosion resistance of TWIP steel welded joints. Moreover, micro-area scanning Kelvin probe (SKP) test has been applied to study the corrosion properties of the micro-area. The relationship between the welding process, microstructure, and corrosion property has thus been established.

## 2. Materials and Methods

### 2.1. Materials and Welding Process

**Figure 1:**
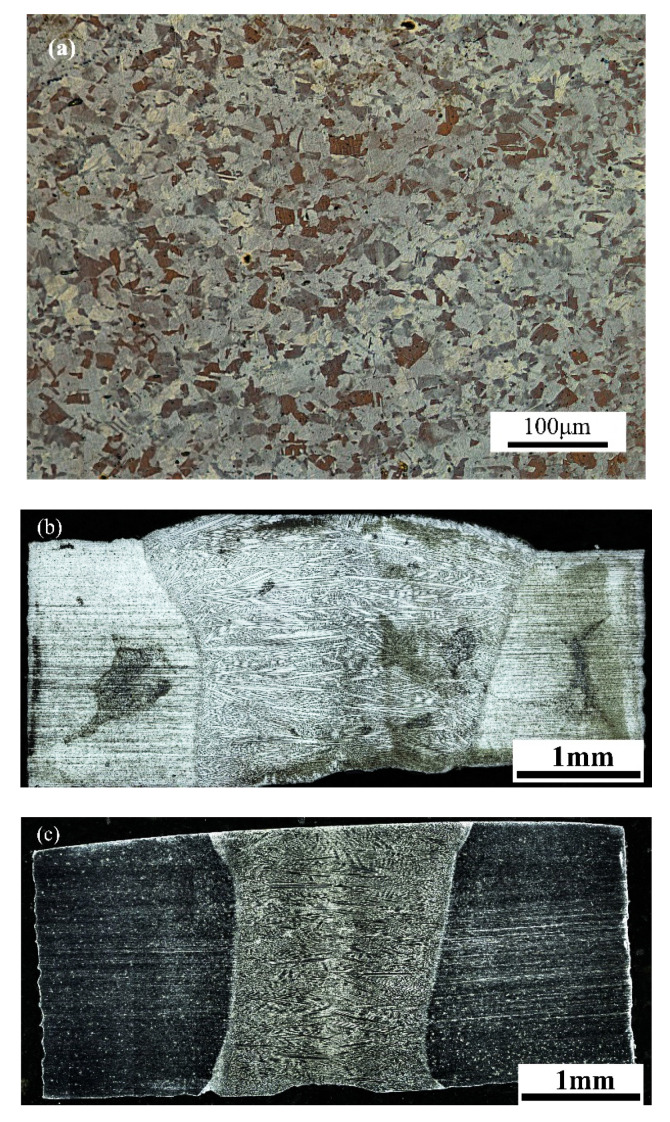
A microstructure of (**a**) the TWIP steel and welded joints, under different welding speeds: (**b**) 1.5 m/min and (**c**) 2.5 m/min.

The as-prepared TWIP steel was used for welding experiments. The sample of TWIP steel and welded joints was ground with Silicon carbide(SiC) sandpaper up to 2000 grit, polished with diamond paste, and etched by using a mixture of nitric acid (2%) and ethanol (98%) to observe the optical microstructure [29]. The chemical composition of TWIP steel is shown in Table 1. The metallographic structure of base metal and welded joints is shown in Figure 1.

**Figure 2:**
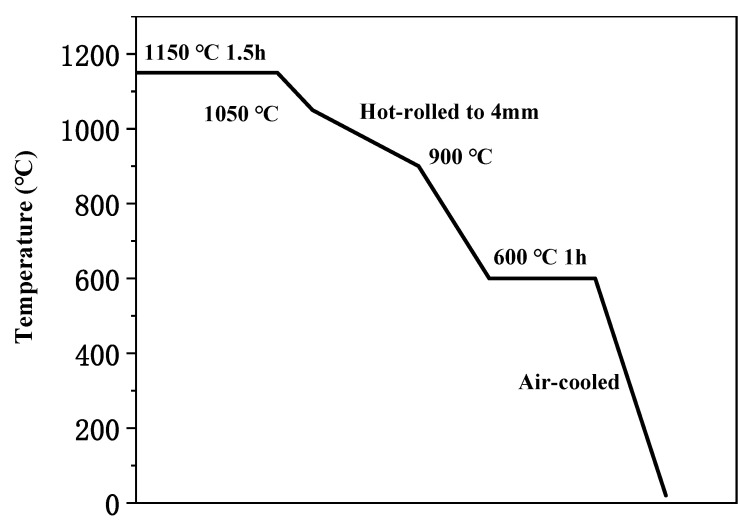
Schematic diagram of thermomechanical control process (TMCP) of TWIP steel.

The chemical composition and metallographic structure are shown in Table 1 and Figure 1, respectively. As shown in Figure 2, TWIP steels were homogenized at 1150 °C for 1.5 h, hot-rolled to 4 mm thickness with initial rolling temperature 1050 °C and finishing temperature above 900 °C, and cooled to a temperature of 600 °C for 1 h. Lastly, all TWIP steel samples were air-cooled to room temperature.

The welding specimens, with the dimensions of 150 mm × 50 mm × 1.5 mm, were processed from the base material of TWIP steel. The length of 50 mm was parallel to the rolling direction, and the welding direction was perpendicular to the rolling direction. The specimens were polished by using an 800 grits sandpaper to obtain a smooth welding interface, which is free of macroscopic defects.

The Adopt TruDiode4006 semiconductor laser was designed and produced by TRUMPF. The IRB4600M2004-type ABB six-axis linkage manipulator was assembled for laser welding. In the rolling direction, laser welding was carried out by using Trudiode4006 semiconductor laser, as the laser which was autogenous, was perpendicular to the specimen surface. The laser power was 3000 W, the spot diameter was 1.5 mm, the focal length was 16 mm, the beam parameter product was 30 mm-mrad, the defocus amount was 14 mm, the focus was below the work piece, and argon gas was purged for protection. The laser welding speed was 1.5 m/min and 2.5 m/min. As shown in Figure 1b,c, it indicates that the welded joints did not exhibit any obvious welding defect, such as lack of penetration, crack, collapse, and edge bite.

### 2.2. Structural and Microstructural Characterization

The phase analysis was carried out at ambient temperature by using micro-area X-ray diffraction (XRD) (Rigaku Smartlab 9 KW, Hypix3000 detector, Cu Kα radiations). The XRD patterns were collected in the 2θ range of 10° to 100° with an incident spot of 0.1 mm. The scanning electron microscopy (SEM) (FEI Quanta 650F, Thermo Fisher Scientific, Hillsboro, OR, USA), equipped with electron backscattered diffraction (EBSD), was used to characterize the microstructure. EBSD analysis was carried out with a scan step of 0.5 μm. The grain size distribution and misorientation between grains were obtained in EBSD data.

### 2.3. Corrosion Properties Characterization

To further explore the corrosion resistance of laser-welded TWIP steel joints, a micro-area SKP test was carried out on the micro-area electrochemical testing device (AMTEK VersaScan, AMTEK, Bowen, PA, USA). The test was performed at ambient temperature in air to obtain the distribution of micro-area volta potential on the surface of a welded joint. The open-circuit potential (OCP) and potentiodynamic polarization curves were measured by electrochemical workstation (AMETEK P4000) in 3.5 wt.% NaCl solution at ambient temperature, using platinum as a counter electrode and a standard calomel electrode (SCE) as reference electrode. The potentiodynamic polarization measurements were carried out after immersion for an hour, while the fluctuations of OCP were less than 5 mv in 10 min. The scanning started from −250 mV vs. OCP toward the anodic potential with a scanning rate of 0.1666 mV/s.

## 3. Results

### 3.1. Structural and Microstructural Analysis

**Figure 3:**
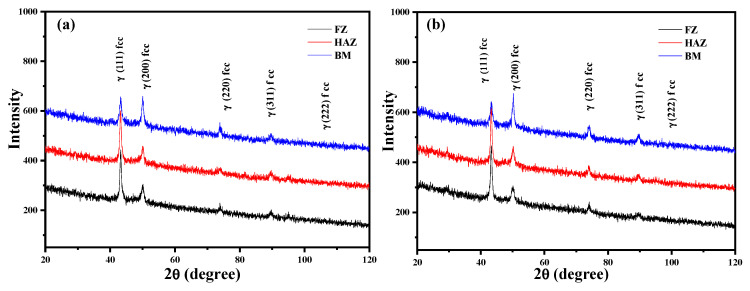
The micro-zone X-ray diffraction (XRD) patterns of welded joints, including base metal (BM), heat-affected zone (HAZ) and fusion zone (FZ), under different welding speeds: (**a**) 1.5 m/min and (**b**) 2.5 m/min.

Figure 3 shows the micro-zone XRD patterns of welded joints, including base metal (BM), heat-affected zone (HAZ), and fusion zone (FZ), under different welding speeds. The phase composition of HAZ and FZ was the same as BM. Moreover, γ (001), γ (200), γ (220), γ (311), and γ (222) diffraction peaks have been observed in all XRD patterns, indicating the austenitic structure of welded joints without any phase transition during melting and cooling processes. In addition, no obvious difference in phase composition of welded samples could be observed at different welding speeds.

**Figure 4:**
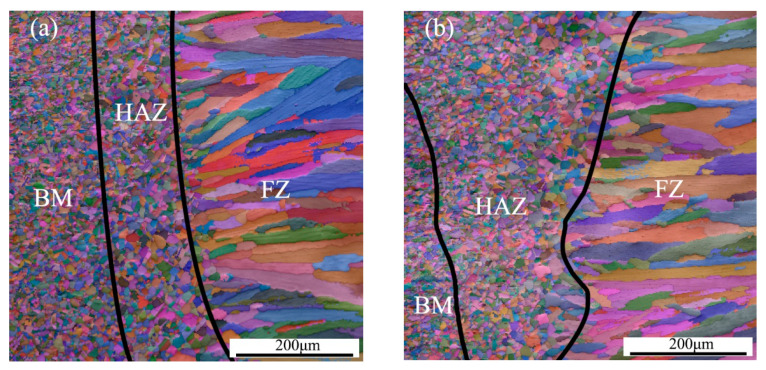
The micrographs of welded joints, including BM, HAZ, and FZ, under different welding speeds: (**a**) 1.5 m/min and (**b**) 2.5 m/min.

The micro-morphology of the welded joints is shown in Figure 4. It can be seen from Figure 4 that the size of FZ in the welded joints was small, and the width was about 100~300 μm. There were fine equiaxed crystals in BM and HAZ, and coarse dendrites in FZ. Moreover, the size of grains in HAZ was larger than those in BM. In general, the welded joints did not exhibit any obvious welding defect, such as lack of penetration, crack, collapse, and edge bite. Moreover, the FZ was attached to the grains of un-melted BM and expanded to the center of the welding pool in the direction of maximum temperature gradient, until coarse dendrites formed after local re-melting. Due to the low thermal conductivity of steel, the HAZ overheated, resulting in serious growth of recrystallized austenite grains and formation of a coarse grain zone [11].

### 3.2. Grain Boundary Characteristics after Welding

**Figure 5:**
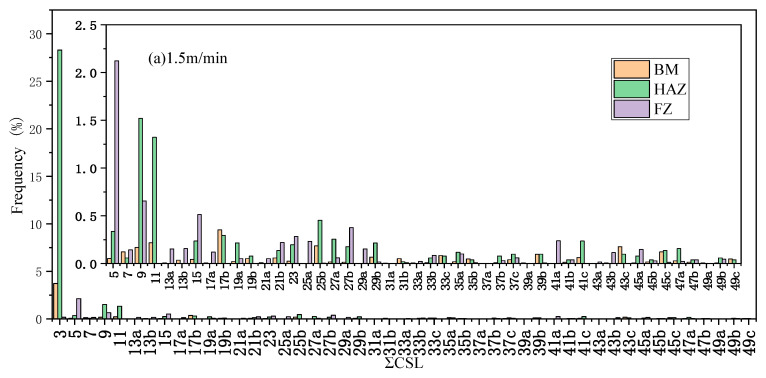

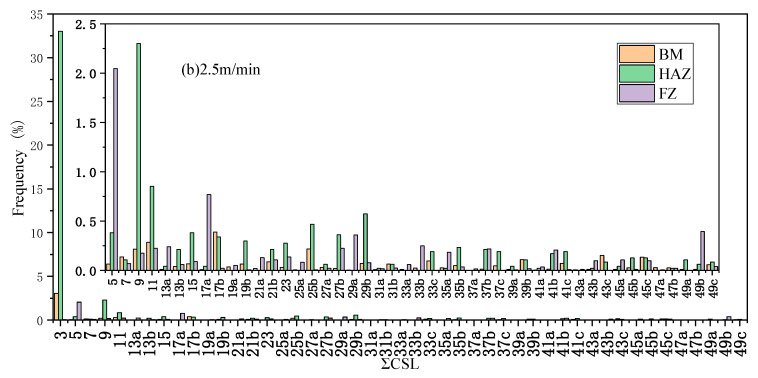
Distribution of ΣCSL boundaries in welded joints: (**a**) 1.5 m/min and (**b**) 2.5 m/min.

Recent research indicates that the grain boundary characteristics, especially coincidence site lattice (CSL) grain boundaries, have a very important influence on the mechanical and corrosion properties of metals [30,31,32,33]. Figure 5 shows the distribution of ΣCSL boundaries in welded joints under different welding speeds. Compared with FZ and BM, more Σ3^n^ grain boundaries could be observed in HAZ. The annealing twins were generally produced in Σ3^n^ grain boundaries of face-centered cubic metals with low layer fault energy. Hence, the low-angle Σ3CSL grain boundaries changed to high-angle grain boundaries (Σ9, Σ27 CSL grain boundaries) along <111> during migration, which could be ascribed to the inconsistency between the lattice of nucleus and substrate [34]. Meanwhile, it can be seen from Table 2 that the frequency of ΣCSL boundaries for FZ in 1.5 m/min was much higher than that in the 2.5 m/min sample.

**Figure 6:**
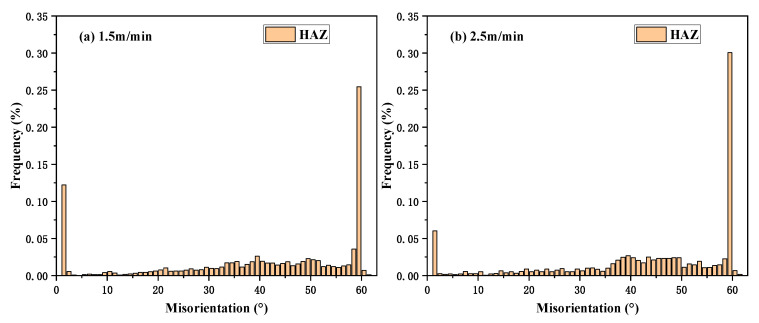
The misorientation distribution of HAZs under the welding speed of (**a**) 1.5 m/min and (**b**) 2.5 m/min.

The misorientation distribution shows that the proportion of 60° orientation difference in HAZ increased, the proportion of 0° orientation difference decreased, and the proportion of 38.74° orientation difference remained unchanged with increasing welding speed (Figure 6). In general, the misorientation is determined by the threshold value of Σ, which implies that the Σ1 CSL grain boundary (sub-boundary) rotated 0°, Σ3 CSL grain boundary rotated 60°, and Σ9 CSL grain boundary rotated 38.74°, along the crystal axis in cubic crystals [35].

**Figure 7:**
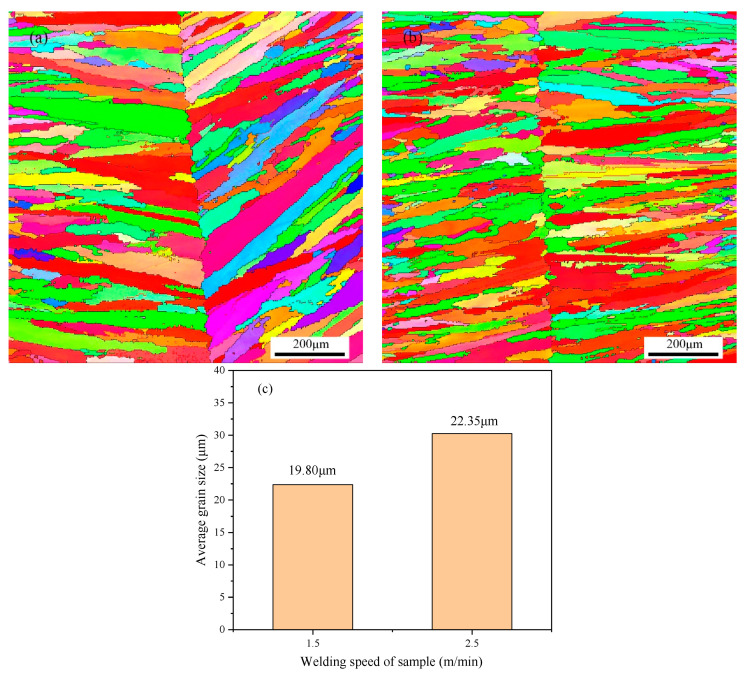
The inverse pole figures (IPF) of FZ under the welding speed of (**a**) 1.5 m/min and (**b**) 2.5 m/min; and (**c**) average grain size of samples.

The inverse pole figures (IPFs) and average grain size of FZ in welded joints are shown in Figure 7. It can be observed that the grain size at 1.5 m/s welding speed was larger than that at 2.5 m/s welding speed (Figure 7c). During the cooling process, the larger secondary dendrite arm consumed the smaller secondary dendrite arm by growing and coarsening, due to the chemical potential difference caused by the difference of curvature and interface energy of crystal. Therefore, the shorter the local solidification time of the molten metal and the faster the cooling rate, the smaller the dendrite grain size [36]. In other words, the increase of welding speed increases the cooling rate of FZ, resulting in a decrease of the average grain size of dendrite grains. The annealing twins inside the dendrites have not been observed, but the dendrites inside the welded joint exhibited large-angle free grain boundaries. One should note that the grain boundaries of FZ grew perpendicular to the <001> direction of the weld center due to the preferential growth of austenite grains, as shown in Figure 7a,b. The growth direction of FZ grains indicated that the preferred orientation was attained under the welding speed of 2.5 m/min.

### 3.3. Potentiodynamic Polarization

**Figure 8:**
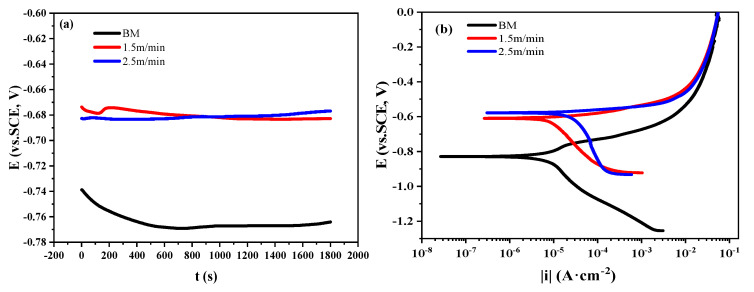
(**a**) Open circuit potentials and (**b**) potentiodynamic polarization curves of BM sample and welded joints.

Potential polarization is an usual way to characterize the corrosion performance of materials. Through the analysis of the electrode process of metals and the fitting of Tafel zones, the corrosion properties of metals can be quantitatively compared. In this paper, a potential polarization test was carried out on TWIP steel welded joints with different welding speeds. As shown in Figure 8a, the OCP of welded joints was higher than the BM sample. The welding speed also rendered a negligible influence on the OCP. The potentiodynamic polarization curves (Figure 8b) indicated that the self-corrosion potential of the welded joints was higher than the BM sample in 3.5 wt.% NaCl solution. Moreover, the fitting results of potentiodynamic polarization curves are summarized in Table 3. Compared with the BM sample, the self-corrosion current density of the welded joints increased by order of magnitude. In addition, the self-corrosion current density of the welded joint under 2.5 m/min was found to be 8.97 × 10^−4^ A·cm^−2^, which was much higher than the welded joint prepared under the welding speed of 1.5 m/min (2.73 × 10^−4^ A·cm^−2^). Therefore, the corrosion resistance of TWIP steel welded joint decreased with the increase of welding speed.

### 3.4. Micro-Area Scanning Kelvin Probe Test

As the most corroded zone, FZ determined the corrosion resistance of the welded joint [27,28]. In order to further explore the influence of laser welding speed on corrosion, a micro-area SKP test was performed on the laser welding joint to characterize the corrosion resistance of each zone.

The voltaic potential difference between the probe and the thin water film on the surface of the corroded metal electrode could be measured by SKP. There is a close relationship between the microstructure of the material surface and volta potential [37,38,39]. In general, the volta potential $V_{kp}$ between the Kelvin probe and the thin liquid film on the corroded metal surface has a linear relationship with the corrosion potential $E_{corr}$ of the corroded metal [40].

(1) $$E_{corr}\;=\;V_{kp}-C$$

Hence, the micro-area SKP technology can be used to characterize the distribution of corrosion potential on the metal surface.

**Figure 9:**
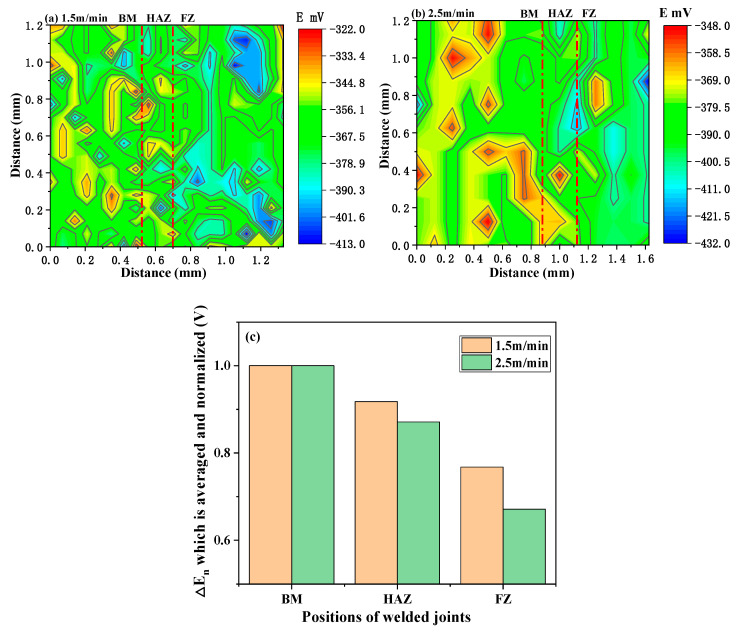
Volta potential distribution of welded joints under the welding speed of (**a**) 1.5 m/min and (**b**) 2.5 m/min; and (**c**) the average surface potential of welded joints.

The volta potential steps between FZ, HAZ, and BM are demonstrated in Figure 9, which demonstrated an increase in the order of FZ, HAZ, and BM. Hence, FZ represented the anode zone due to the lowest surface potential in welded joint, whereas HAZ and BM constituted the cathode zone in a primary corrosive cell. In order to quantitatively compare the surface potential of FZ, HAZ, and BM, $\Delta E_{n}$ was introduced.

(2) $$\Delta E_{n}\;=\;V_{n}-V_{0}$$

where $V_{n}$ is the volta potential at each position, and $V_{0}$ represents the lowest volta potential on the surface of samples. The $\Delta E_{n}$ for FZ, HAZ, and BM were averaged and normalized based on the surface potential of BM to obtain Figure 9c. For the sample of 1.5 m/min, the potential difference between BM and HAZ was larger than that of 2.5 m/min. The potential difference between HAZ and FZ for the sample of 1.5 m/min was smaller than that for the sample of 2.5 m/min.

Considering that the $\Delta E_{n}$ for BM of the two samples with different welding speed were consistent, as the normalized result was 1, the $\Delta E_{n}$ for HAZ in the sample of 1.5m/min was slightly higher. Furthermore, for FZ, $\Delta E_{n}$ for the 2.5 m/min sample was significantly lower, which means that the FZ in 2.5 m/min sample had the worst corrosion resistance.

It can be seen from Figure 9 that the corrosion resistance of the BM in laser welded joints was the best, followed by HAZ, and that of FZ as the worst. In general, the corrosion of laser welded joints often started from FZ. Therefore, the focus is on the relationship between microstructure and corrosion properties in FZ.

The comparison of Figure 4c and Figure 9c reveals a positive correlation between the number of Σ 3^n^ CSL grain boundaries and surface potential of FZ. The number of Σ3^n^ CSL grain boundaries in FZ for 1.5 m/min sample was higher than that for 2.5 m/min sample. In general, there were the least Σ3^n^ CSL grain boundaries in FZ in the 2.5 m/min sample, and it showed the worst corrosion resistance. One should note that the Σ3^n^ CSL grain boundaries were beneficial to improve the corrosion resistance of the TWIP steel [40]. This was because the Σ3^n^ grain boundaries exhibited high resistance to grain boundary failure [29,30,31] and the corrosion resistance could be improved by increasing the frequency of CSL grain boundaries and controlling the continuity of grain boundaries [32]. Therefore, the large distribution of CSL grain boundaries interrupted the connectivity of random grain boundaries, which effectively prevented the continuous corrosion. The higher frequency of CSL grain boundaries with low Σ value (Σ ≤ 27) resulted in better corrosion resistance.

On the other hand, the grain size of FZ in 1.5 m/min sample was less than that in the 1.5 m/min sample (Figure 7c). During the growth of grains, random large-angle grain boundaries will increase, while sub-grain boundaries will decrease relatively, whereas the coarse grain structure has an adverse effect on the corrosion resistance. This means that the larger the grain, the worse the corrosion resistance [41,42,43]. Therefore, the corrosion resistance of the 2.5 m/min sample was weaker, which was consistent with the electrochemical results (Figure 8b). In general, the superior corrosion resistance of the 1.5 m/min sample could be ascribed to the higher frequency of CSL grain boundaries and finer grains in FZ.

## 4. Conclusions

In summary, the influence of laser welding rate on TWIP steel has been investigated. The following conclusions can be drawn from the current results:

1. The preferred orientation of grains and the grain size in FZ increased with the increase of welding speed. However, the CSL grain boundary of FZ decreased with increasing welding speed.
2. The surface potential of FZ, HAZ, and BM in each welded joint sequentially increased. The corrosion resistance of the BM in laser-welded joints was the best, followed by HAZ, and that of FZ as the worst.
3. The 1.5 m/min sample rendered better corrosion resistance, due to the higher frequency of CSL grain boundaries and finer grains in FZ.

This study provides ideas for research on the corrosion properties of TWIP steel laser-welded joints under specific conditions, and provides a reference for the TWIP steel laser welding process under chloride ion corrosion environment. Subsequent studies could focus on the corrosion behavior and mechanism of TWIP steel laser welded joints in different corrosive environments.

## Figures and Tables

**Table 1:** The chemical composition of as-prepared twinning-induced plasticity (TWIP) steel (wt.%).

Element	C	Si	Mn	Al	P	S	RE	Cr	Fe
TWIP steel	0.6	0.3	18	1.5	<0.01	<0.01	<0.01	0	Bal.

**Table 2:** The frequency of Σ3^n^ CSL.

Sample	BM	HAZ	FZ
1.5 m/min	3.92%	30.25%	1.27%
2.5 m/min	3.33%	35.74%	0.47%

**Table 3:** The potentiodynamic polarization results of the BM sample and welded joints.

Sample	Ecorr (mV vs. SCE)	Icorr (A·cm^−2^)
1.5 m/min	−609.18	2.73 × 10^−4^
2.5 m/min	−592.95	8.97 × 10^−4^
BM	−829.01	3.42 × 10^−5^
